# Impact of met-haemoglobin and oxidative stress on endothelial function in patients with transfusion dependent β-thalassemia

**DOI:** 10.1038/s41598-024-74930-3

**Published:** 2024-10-25

**Authors:** Maha Abubakr Feissal Rabie, Sanaa A. El Benhawy, Inas M. Masoud, Amal R. R. Arab, Sally A. M. Saleh

**Affiliations:** 1 Department of Basic Science, Pharos University in Alexandria, Canal El Mahmoudia Street, Beside Green Plaza Complex, Alexandria, Egypt; 2 Radiation Sciences Department, Medical Research Institute, Alexandria University, Alexandria, Egypt; 3 Department of Pharmacology and Therapeutics, Faculty of Pharmacy, Pharos University in Alexandria, Alexandria, Egypt; 4 Department of Applied Medical Chemistry, Medical Research Institute, Alexandria University, Alexandria, Egypt; 5 Department of Clinical Haematology, Medical Research Institute, Alexandria University, Alexandria, Egypt

**Keywords:** Transfusion depended β-thalassemia, EASIX, Oxidative stress, Met-Hb, Malondialdehyde, Diseases, Health care, Medical research, Pathogenesis

## Abstract

Transfusion dependent β-thalassemia is a genetic blood disorder characterized by chronic anaemia. Blood transfusion is lifesaving but comes at a cost. Iron overload emerges as a prime culprit as a free radicals damage endothelial cells. Chronic anaemia further disrupts oxygen delivery, exacerbating the oxidative stress. Increased levels of met-haemoglobin and malondialdehyde compromise endothelial function. This research sheds light on the impact of met-haemoglobin and oxidative stress on endothelial function in 50 patients with transfusion dependent β-thalassemia major compared to 50 healthy individuals as control. Blood samples were collected & subjected to CBC, biochemical analysis including creatinine, ferritin, CRP, LDH, and HCV antibodies. Oxidative stress was assessed using met-haemoglobin & malondialdehyde. Endothelial dysfunction was evaluated by endothelial activation and stress index (EASIX). EASIX, met-haemoglobin and malondialdehyde were significantly increased in patients (1.44 ± 0.75, 2.07 ± 0.2, 4.8 ± 0.63; respectively) compared to the control (0.52 ± 0.24,0.88 ± 0.34,0.8 ± 0.34; respectively). Significant strong positive correlation was found between EASIX and met-haemoglobin, malondialdehyde, serum ferritin and CRP (*P* = 0.00, *r* = 0.904, *P* = 0.00, *r* = 0.948, *P* = 0.00, *r* = 0.772, *P* = 0.00, *r* = 0.971; respectively. Met-haemoglobin as well as EASIX should be routinely estimated to assess endothelial function especially before the decision of splenectomy. Antioxidant drugs should be supplemented.

## Introduction

β-thalassemia is a major monogenic disease with reduced or absent β globin genes^1^. The unpaired α-globin gene results in chronic haemolysis and ineffective erythropoiesis necessitating lifelong blood transfusion. This exposes the patients to many complications namely; chronic anaemia, iron overload, oxidative stress, hypercoagulability and platelet activation in which damaged endothelial cells will be the end sequence^2,3^.

The normal endothelium serves as a critical regulator of vascular homeostasis, maintaining a delicate equilibrium between diverse physiological processes. These include vasomotor function (dilation and constriction), vascular smooth muscle cell growth and death, a balanced release of pro- and anti-inflammatory mediators, and the delicate interplay of oxidative stress and antioxidant defense^4^. Chronic haemolysis in transfusion-dependent β-thalassemia (TDT) is a key driver of inflammation as red blood cells destruction releases inflammatory mediators that can lead to oxidative stress, immune system activation, and iron overload^5^. A relationship between inflammatory biomarkers and thalassemia has been recently reported^6^. Inflammation makes endothelium to undergo multiple functional modifications that are adaptive but become harmful when exceeding, leading to microvascular dysfunction and multiorgan failure^7^. Moreover; the endothelial dysfunction is caused by the interplay of adhesion molecules and inflammatory cytokines such soluble intercellular adhesion molecule and E-selectin^8,9^.

Oxidative stress in TDT patients is caused mostly by excess non-transferrin bound iron, a powerful free radical that destroys organs, DNA and mitochondria. TDT patients are likely to have simultaneous intravascular haemolysis when subjected to oxidative stress which causes haemoglobin oxidation and the formation of met-haemoglobin (Met-Hb)^10,11^.

Moreover, chronic anaemia by itself can lead to increased reactive oxygen species (ROS) production as the body compensates for low oxygen level which directly damages endothelial cells threatening vasodilation by impairing nitric oxide (NO) bioavailability, a potent vasodilator and key regulator of endothelial function^12,13^. ROS also trigger auto-oxidation of haemoglobin, accelerating the conversion of ferrous (Fe²⁺) to ferric (Fe³⁺) state within the haemoglobin molecule, resulting in excessive Met-Hb formation (more than 1%). Met-Hb is an abnormal form of haemoglobin that incapable of delivering oxygen to tissues as ferric iron cannot reversibly bind oxygen causing left shift in oxygen dissociation curve and eventual tissue hypoxia particularly in the vascular endothelium leading to endothelial dysfunction^14^. Additionally, Met-Hb’s pro-oxidant properties can trigger oxidative stress causing structural and functional changes in the endothelium^15^. These changes include increase oxidative damage to endothelial cells and impaired NO bioavailability^6^, as Met-Hb scavenges it, which results in impaired vasodilation, decreased blood flow and ultimately endothelial dysfunction with upregulation of inflammatory markers and release of interleukin 1 beta, neutrophil activation and degranulation of Weibel-Palade bodies^16,17^.

Chronic blood transfusion in TDT exposes these patients to the effect of iron overload, which damages tissues and triggers an inflammatory response by generating the formation of ROS that directly oxidise haemoglobin, contributing to elevated Met-Hb levels. In the presence of chronic haemolysis, this causes oxidative damage to the endothelium and activation of inflammatory pathways. Chronic transfusion also exposes them to a bioactive product in blood bags resulting in pro-inflammatory status that in turn produces vascular inflammation and endothelial dysfunction^6,18^. Increased platelet count in these patients can be considered as a marker of systemic inflammation which is a feature of endothelial dysfunction^19,20^. Finally certain medications used in transfusion therapy such as some antibiotics can induce Met-Hb formation, further exacerbating the condition in TDT patients^8,21,22^. To the best of our knowledge, no previous studies have thoroughly investigated the impact of oxidative stress mediated by Met-Hb on endothelial function using EASIX in TDT. Consequently, the aim of our research was to assess the impact of met-haemoglobin and oxidative stress on endothelial function in patients with transfusion dependent β-thalassemia major.

## Patients and methods

### Ethical information

The study was approved by the Ethics Committee of The Medical Research Institute (MRI) - Alexandria University on 26 December 2023 - Approval serial number: E/C. S/N. R14/2023. All methods were performed in accordance with the declaration of Helsinki. All participants were freely volunteer for the study with informed written consents prior to their participation. According to the Ethical Guidelines of MRI, Alexandria University the study guarantees the privacy of any personal information regarding the patients’ data.

### Patients and study design

Our study was carried out in MRI hospital in Alexandria, Egypt, from December 2023 to March 2024. It included fifty Egyptian TDT patients who were initially diagnosed by Hb electrophoresis, PCR for β-globin gene and on iron chelating therapy. Fifty healthy controls matched by age (range 18–35 years) and sex were also included. All cases of non-transfusion dependent β-thalassemia (NTDT), congenital met-haemoglobinemia, unstable haemoglobin, sickle cell diseases or smokers were excluded.

Each patient gave five millilitres of blood; two of them were collected in a VACUETTE^®^ EDTA tube for complete blood count (CBC) carried out on a Siemens ADVIA^®^ 2120i Haematology Analyser. Microscopic inspection was also done to confirm the differential count. Three millilitres were collected in a VACUETTE^®^ Z serum sep clot activator tube for creatinine, ferritin, C-reactive protein (CRP), lactate dehydrogenase (LDH) and assessed by the HITACHI AUTOMATIC ANALYZER COBAS 6000, lot (52520500). Hepatics-C virus positivity was detected by using anti-HCV antibodies (anti-HCV) CTK BIOTECH ELISA kit. Oxidative stress was assessed using malondialdehyde (MDA) and Met-Hb.

### Estimation of serum Malondialdehyde (MDA) concentration (nmol/mL): -

Thiobarbituric acid and MDA react in acidic media at 95 °C for 30 min to create a reactive product. Colorimetric measurements of the pink product’s absorbance at 534 nm were done in accordance with the manufacturer’s methodology (Biodiagnostics, Egypt).

### Estimation of serum met-haemoglobin (Met-Hb) level (µg/mL): -

By using human met-haemoglobin BIOMATIK ELISA kit {Cat#EKC34599}.

Endothelial dysfunction was evaluated by calculating endothelial activation and stress index (EASIX) = (LDH {U\L}x creatinine {mg\dL}/thrombocytes {10^9 cells /L})^23,24^.

### Statistical analysis

The Kolmogorov-Smirnov test was used to assess the normality of continuous data. Studied parameters were presented as mean and standard deviation. Independent-samples t test was used for mean comparison between studied groups. Odds ratios (OR) with corresponding 95% confidence intervals (CI) were calculated. Pearson correlation was used to test correlation among the different studied parameters. Statistical significance was assumed at a level of P values < 0.05. Statistical calculations were performed using SPSS 25.0 for Windows (SPSS, Inc., Chicago, IL, USA).

## Results and discussion

The endothelium has important functions namely; control of coagulation, fibrinolysis, vascular tone and immune response^25^. Normal cellular metabolism generates ROS as a by-product of the mitochondrial electron transport chain. These highly reactive molecules are constantly neutralized by the body’s antioxidant defence system to maintain a state of redox homeostasis. However, an imbalance between ROS production and antioxidant capacity can lead to a condition known as oxidative stress^26^.

Table 1 shows that haemoglobin and neutrophils / platelets ratio (NPR) were significantly decreased in TDT patients compared to the control. No significant difference was detected as regards neutrophils, lymphocytes and neutrophils/ lymphocytes ratio (NLR) while the remaining parameters were significantly higher in our patients. These are concordant with Ayyash et al.,^27^. The results of Aldwaik R et al.,^28^ was matched with our finding as regards the haemoglobin mean value (8.4 ± 1.4 g/dl) in TDT patients. Solangi et al.,^29^ results were matched with us as *regards* reduction in Hb and lymphocyte levels but were different for platelet count which was elevated in our study. We can explain the higher platelets in our patients by that 64% of them were splenectomised. Although it is well-known that platelet counts often rise after splenectomy as reported by Sande et al.,^30^ yet no systematic study has examined how much of an increase can be anticipated and when exactly these counts will decrease post-splenectomy.

**Table 1:** Comparison of CBC parameters between TDT patients and control.

	TDT patients (*n* = 50) Mean ± S. D	Control (*n* = 50) Mean ± S. D	*P*
Hb (g/dL)	8.2 ± 0.97*	12.76 ± 0.67	0.000
WBCs (UL*10^3)	9.58 ± 3.86*	6.87 ± 0.97	0.000
Neutrophils (*10^3/mL)	54.08 ± 8.8	54.06 ± 4.22	0.988
Lymphocytes (UL*10^3)	34.9 ± 6.65	35.86 ± 4.12	0.388
Platelets (*10^3/mL)	451 ± 177*	279 ± 64.54	0.000
NLR	1.64 ± 0.58	1.53 ± 0.27	0.222
PLR	13.77 ± 7*	7.79 ± 1.6	0.000
NPR	0.14 ± 0.05*	0.20 ± 0.05	0.000

*Hb*: Haemoglobin, *WBCs*: White blood cells, *NLR*: Neutrophils/ Lymphocytes ratio, *PLR*: Platelets/ Lymphocytes ratio. *NPR*: Neutrophils / Platelets ratio. *P* value for comparing between TDT patients and control group.

*Statistically significant at *p* ≤ 0.05.

Table 2 demonstrates that TDT patients had a significantly higher Met-Hb that exacerbates the oxidative stress which in turn has a negative impact on the endothelial function. Our finding is aligned with a study conducted by Vardhanabhuti et al.,^11^ who stated that the high level of Met-Hb in TDT patients might be due to a surplus release of unstable haemoglobin resulting from excessive red blood cell turnover. Organ failure and disease severity are exacerbated by increased levels of circulating cell-free haemoglobin in haemolytic and inflammatory disorders. Multiple investigations into haemoglobin’s role in tissue damage, have been studied but the exact signalling pathways behind haemoglobin-mediated endothelial dysfunction in various organs remains incomplete^16^.

**Table 2:** Comparison of biochemical and endothelial markers between TDT patients and control.

	TDT patients (*n* = 50) Mean ± S. D	Control (*n* = 50) Mean ± S. D	*P*
Met-haemoglobin (µg/mL)	2.07 ± 0.2*	0.88 ± 0.34	0.000
Malondialdehyde (nmol/mL)	4.8 ± 0.63*	0.80 ± 0.34	0.000
EASIX	1.44 ± 0.75*	0.52 ± 0.24	0.000
Ferritin (ng/mL)	2921 ± 1355*	96 ± 23	0.000
Creatinine(mg/dL)	1.09 ± 0.25*	0.64 ± 0.17	0.000
LDH (U/L)	510 ± 147*	215 ± 64	0.000
CRP (mg/dL)	4.24 ± 2.14*	2.24 ± 0.63	0.000

*EASIX*: Endothelial activation & stress index, *LDH*: Lactate dehydrogenase, *CRP*: C-Reactive Protein, *P* value for comparing between TDT patients and control group. *Statistically significant at *p* ≤ 0.05.

We found that TDT patients had a significantly higher serum MDA “A pro-oxidant biomarker formed by the lipid peroxidation of polyunsaturated fatty acids in cells caused by iron overload-induced oxidative stress” and ferritin levels than control. Basu et al.,^31^ metanalysis results are in agreement with our findings. In addition, Neaimy et al.,^32^ showed that individuals with β thalassemia had lower total antioxidant capacity values and a higher MDA levels. Al-Hakeim et al.,^33^ stated that increased MDA in thalassemia could be due to vulnerability of these patients to tissue injury caused by high oxidative stress. Moreover, formation of MDA is independent of excess non-labile iron concentration indicating that different mechanisms are involved in injury caused by the labile iron and the formation of oxidation end products. An increased risk of cellular and molecular damages caused by oxidative stress and its associated diseases, as well as ineffective anti-oxidant defence systems for β-thalassemia patients was reported by Fatima T et al.^34^.

Although serum ferritin (SF) has been demonstrated to be a marker of iron overload, its threshold varies and larger levels are classed differently highlighting the problem of using it generally to predict disease outcomes^35^. In our study SF levels in TDT patients ranged from (772–6849 ng/mL) with a mean value of (2921 ± 1355 ng/mL). Shah et al.,^36^ stated that organ damage, greater risk of cardiac events, hepatic problems and increased mortality are among the negative outcomes linked to iron overload which is indicated by SF levels exceeding 1000 µg/L. The wide range of SF in these patients is attributed to many factors i.e. compliance to iron chelation therapy, presence of hepatitis C virus (HCV) infection, splenectomy as well as different haptoglobin polymorphism^37^. SF impact is felt by patients at various points in their lives due to the fact that TDT is a chronic condition with lifelong blood transfusion. Garbowski et al.,^38^ illustrated that reduction in SF was coupled with increased erythroid iron incorporation and higher bone marrow erythroid production, thereby decreasing transfusion requirements and promoting the redirection of bounded and free iron to the liver.

In the present study EASIX was significantly elevated in TDT patients compared to control which works as a novel diagnostic tool for endothelial dysfunction. Luft, T. et al.,^39^ found that the levels of EASIX serve as surrogate indicators when determining the amount of endothelial impairment. Our finding is in accordance with Scioli et al.,^40^ who showed that higher EASIX reflects endothelial dysfunction that could be attributed to enhanced oxidative stress.

**Fig. 1:**
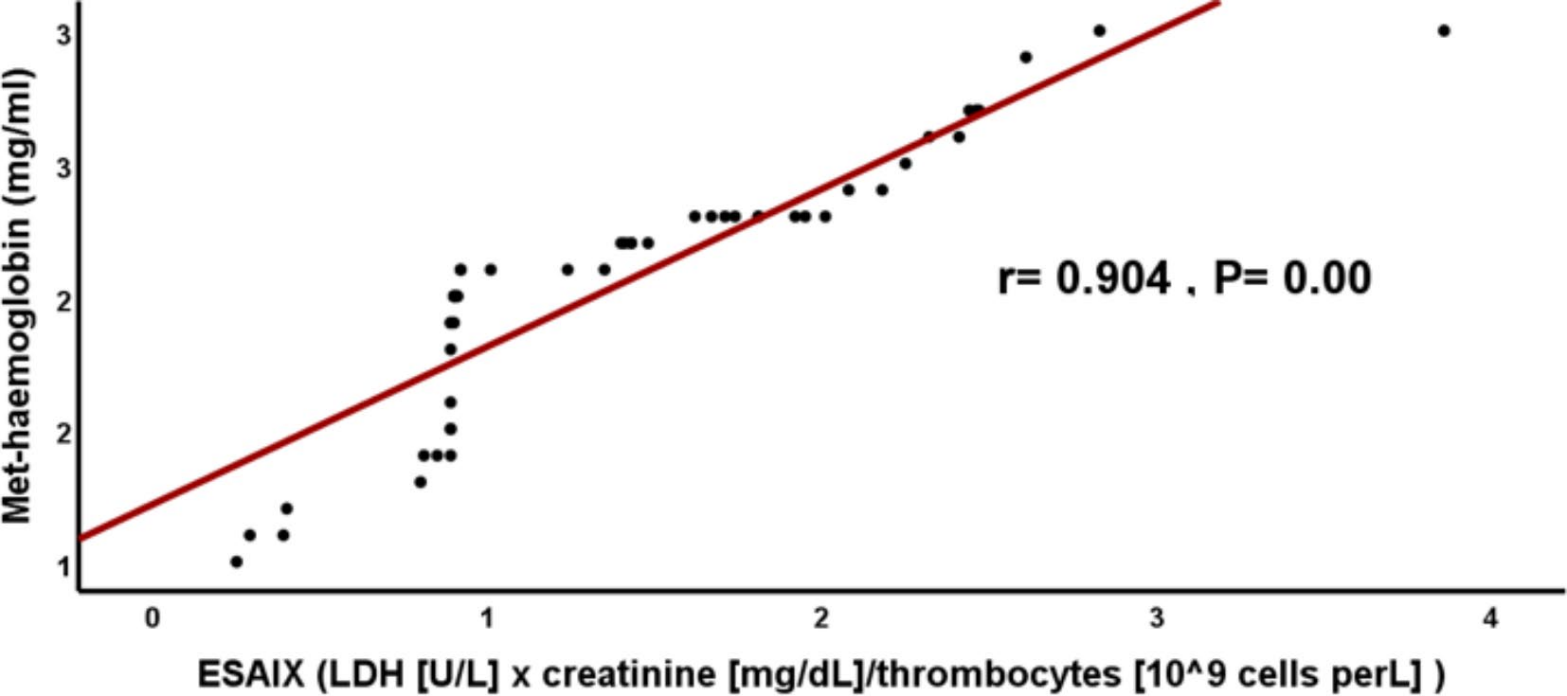
Correlation between EASIX and serum Met-Hb among TDT patients. EASIX: Endothelial activation & stress index, *r*: person correlation coefficient. *: Statistically significant at *p* ≤ 0.05.

**Fig. 2:**
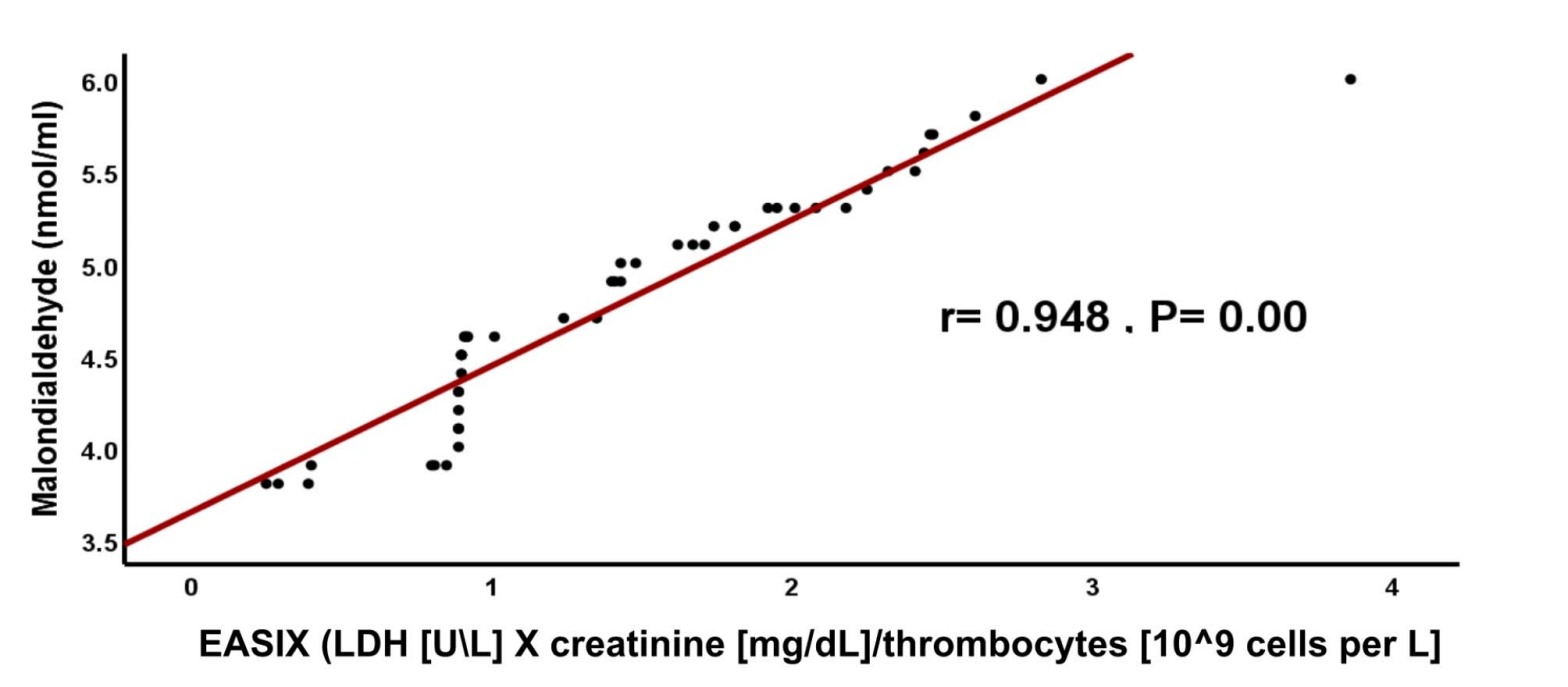
Correlation between EASIX, and serum malondialdehyde among TDT patients. EASIX: Endothelial activation & stress index, *r*: person correlation coefficient. *: Statistically significant at *p* ≤ 0.05.

**Fig. 3:**
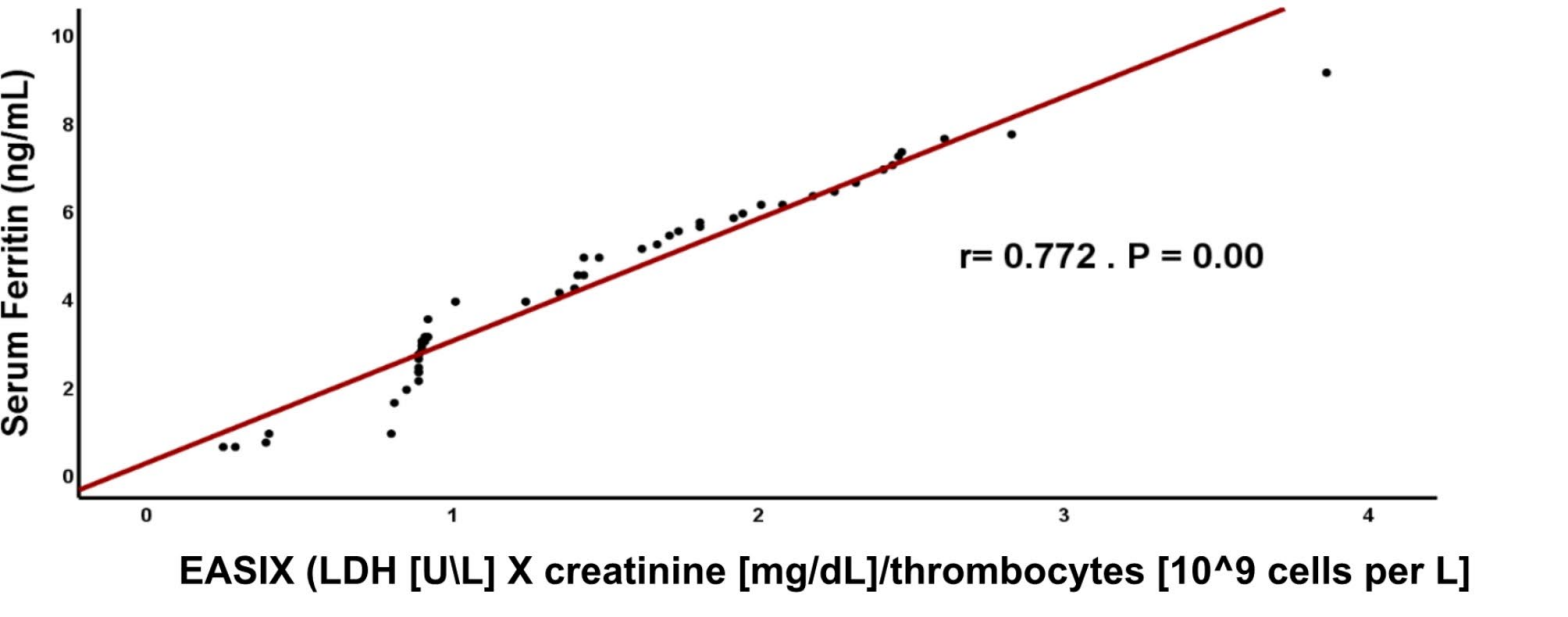
Correlation between EASIX, and serum Ferritin among TDT patients. EASIX: Endothelial activation & stress index, r: person correlation coefficient. *: Statistically significant at *p* ≤ 0.05.

**Fig. 4:**
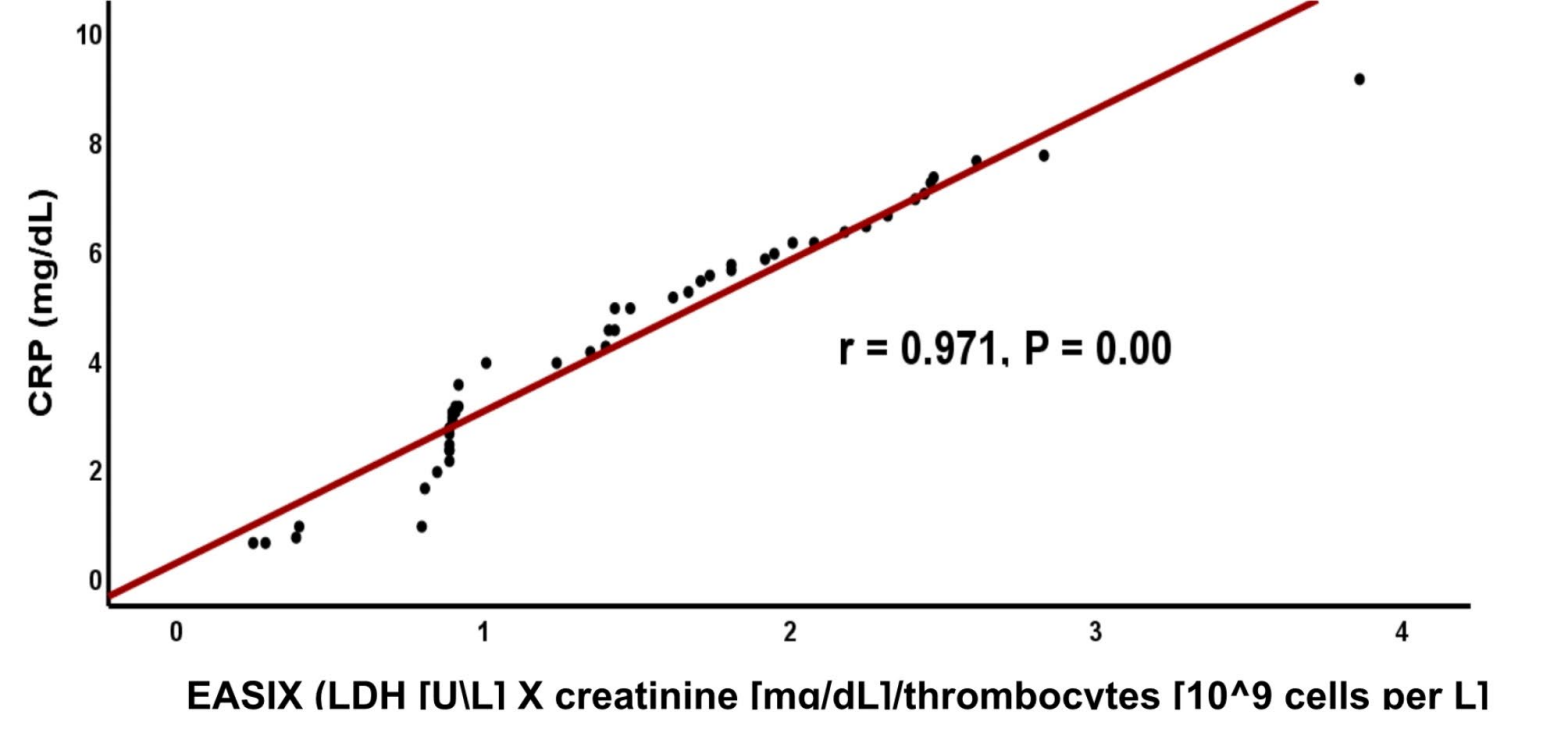
Correlation between EASIX, and serum CRP among TDT patients. EASIX: Endothelial activation & stress index, *CRP*: C-reactive protein, *r*: person correlation coefficient. *: Statistically significant at *p* ≤ 0.05.

 We observed a significant positive correlation between EASIX, Met-Hb, MDA, SF and CRP (r =0.904, r = 0.948, r= 0.772 and r= 0.971; respectively) among TDT patients (Figs. 1, 2, 3, 4). These findings eliminate the interaction between oxidative stress and the previously indicated factors that implicate inflammation in causing dysfunction of the vascular endothelium. This is supported by a strong positive connection between CRP, a biomarker of inflammation, and EASIX. According to Liu et al.^9^ Met-Hb significantly increases chemokine and cytokine production as well as the expression of cell adhesion molecules through NF-kappa B. Met-Hb-induced endothelial cell activation during infections, hemolysis, or met-haemoglobinemia is of paramount clinical importance.

Thalassemia by itself significantly amplifies the effects of oxidative stress^32^. TDT patients who have a co-infection with HCV face a twofold effect of oxidative stress, as HCV infection exacerbates the activation of free radical production, disrupts lipid metabolism and cholesterol transport^41^. The progression of HCV infection is linked more to oxidative stress, as it causes cellular hypoxia, defective angiogenesis, and an ineffective immune response^42^.The circulating levels of inflammatory cytokines are increased by HCV infection, which also has an impact on endothelial function^43^.

Table 3 shows that HCV positive TDT patients experience a more severe degree of oxidative stress (significantly higher Met-Hb, MDA and EASIX) compared to their HCV-negative counterparts. Iron overload, resulting in excessive production of ROS due to the accumulation of unbound iron in hepatocytes, as well as viral hepatitis, can lead to chronic inflammation, liver fibrosis and cirrhosis in TDT patients^41,44^. Our study demonstrated that Hb, WBCs, neutrophils, platelets, NLR and PLR were significantly lower in HCV positive TDT patients. It is commonly believed that autoimmune destruction, hypersplenism, antiviral treatment and reduced thrombopoietin level are the causes of cytopenias in HCV infection^45^. Autoimmune haemolytic anaemia is primarily linked to treated HCV positive patients, but it has also been observed in untreated patients. Ribavirin, for instance, can cause a decrease in the amount of intracorpuscular adenosine triphosphate and make them more susceptible to oxidative harm and extravascular haemolysis^46,47^.

**Table 3:** Comparison of HCV negative & positive status among TDT patients.

	HCV Negative TDT patients (*n* = 32) Mean ± S. D	HCV Positive TDT patients(*n* = 18) Mean ± S. D	*P*
Met-haemoglobin (µg/mL)	1.97 ± 0.47	2.26 ± 0.49*	0.043
Malondialdehyde (nmol/mL)	4.6 ± 0.6	5.1 ± 0.61*	0.018
EASIX	1.07 ± 0.44	2.1 ± 0.74*	0.001
Hb (g/dL)	8.54 ± 0.92	7.6 ± 0.75*	0.001
WBCs (UL*10^3)	10.98 ± 3.62	7.1 ± 2.93*	0.000
Neutrophils (*10^3/mL)	57.0 ± 9.0	48.8 ± 5.4*	0.001
Platelets (*10^3/mL)	535 ± 161	304 ± 89*	0.000
NLR	1.79 ± 0.58	1.38 ± 0.49*	0.013
PLR	16.74 ± 6.7	8.5 ± 3.5*	0.000

*EASIX*: Endothelial activation & stress index, *Hb*: Haemoglobin, *WBCs*: White blood cells, *NLR*: Neutrophils/ Lymphocytes ratio, *PLR*: Platelets/ Lymphocytes ratio, *P* value for comparing between TDT patients HCV infection status. *: Statistically significant at *p* ≤ 0.05.

Among HCV patients Kedia et al.;^48^ explained that leucopenia or neutropenia could be caused by hypersplenism while other researchers^49^ proposed that the aetiology might be autoimmune in nature. According to Abou El Azm et al.,^50^ direct bone marrow involvement may be the underlying reason. Additionally, Aref et al.,^51^ mentioned that caspase 10 activation and enhanced neutrophil apoptosis were seen. Furthermore, Sulkowski et al.,^52^ elucidated that neutropenia is a prevalent occurrence in HCV-infected individuals undergoing treatment being an adverse impact of medication.

TDT can impact both PLR and NLR, although the exact mechanisms and clinical implications are still being explored. TDT patients experience chronic haemolysis that can lead to increased breakdown products in the bloodstream, triggering inflammation and increasing bone marrow activity in order to compensate for red blood cell loss, potentially affecting other blood cell lines leading to a possible decrease in PLR^53^. Chronic viral hepatitis is characterised by a persistent inflammatory response in which the PLR and NLR are connected with the course and prognosis of viral hepatitis-related complications^54^.

Meng et al.,^55^ studied PLR in different HCV infection stages and found that there was a decrease in PLR in chronic HCV infected patients than control, which is matched with our results as regards HCV positive and negative TDT patients. This could be explained by that HCV might indirectly suppress the bone marrow, leading to a decrease in platelet production also chronic inflammation associated with HCV could increase platelet activation and consumption.

The decision for splenectomy in thalassemia requires careful consideration of potential benefits and drawbacks regarding disease severity, timing of splenectomy and individual variations in addition to its impact on oxidative stress and endothelial function^56^. Table 4 shows a significantly higher value of Met-Hb, MDA, CRP and EASIX among splenectomised TDT patients. The impact of splenectomy on oxidative stress in thalassemia patients is a topic of some debate. While the spleen plays a role in the body’s antioxidant defence system, researches haven’t shown a definitive link between splenectomy and increased oxidative stress. The effect of splenectomy on serum total antioxidant capacity (TAC) in β--thalassemia patients has been studied by Talat et al.,^57^ who found a lower TAC levels in splenectomised children but the difference wasn’t statistically significant. It seems that splenectomy is highly linked to endothelial dysfunction as shown by our study where EASIX was significantly higher among splenectomised TDT patients.

**Table 4:** Comparison between non-splenectomised and splenectomised TDT patients.

	Non-Splenectomised TDT patients (*n* = 18) Mean ± S. D	Splenectomised TDT patients (*n* = 32) Mean ± S. D	*P*
Met-haemoglobin (µg/mL)	1.56 ± 0.34	2.37 ± 0.28*	0.000
Malondialdehyde (nmol/mL)	4.1 ± 0.25	5.2 ± 1.5*	0.000
CRP (mg/dL)	1.99 ± 0.87	5.5 ± 1.5*	0.000
EASIX	0.76 ± 0.24	1.83 ± 0.66*	0.000

*CRP*: C-Reactive Protein, *EASIX*: Endothelial activation & stress index, *P* value for comparing between TDT patients with normal and enlarged spleen. *: Statistically significant at *p* ≤ 0.05.

Additionally, splenectomy increases platelet count, haematocrit, the durability of procoagulant-damaged erythrocytes and platelet-derived microparticles in patients with chronic haemolysis^56,58^. The relationship between endothelial dysfunction and splenectomy in TDT was investigated by Aggeli et al.,^59^ who found that β-thalassemia major patients may experience endothelial dysfunction due to persistent inflammation and splenectomy might make the problem worse. The study adds to our knowledge of how splenectomy may affect variables that cause endothelial dysfunction.

For a deeper exploration of the clinical implications of our results and how they might influence treatment strategies for TDT patients, we can shed light on that iron overload leads to oxidative damage in vital organs. Chelation therapy, which aims to sequester redox-active iron, is a used strategy to mitigate oxidative stress in these patients^60^. Endothelial dysfunction, inflammation, thrombosis, leukocyte adhesion and mitochondrial damage are all promoted by oxidative-stress-induced ROS generation^61^. Oxidative stress and endothelial dysfunction are also associated in the pathophysiology of various vascular and metabolic illnesses including peripheral vascular disease, stroke, heart disease and diabetes. Thus, understanding how Met-Hb disrupts endothelium function is essential for designing effective treatments for these illnesses^62^. Strategies to decrease Met-Hb levels, such as administering high doses of ascorbic acid or methylthioninium chloride (new methylene blue), could be applied to TDT patients^63^. TDT emphasises the importance of specific components in oxidative status; N-acetyl cysteine and vitamin E are particularly effective antioxidant supplements among them^64^.

### Limitation of the study

The study included only transfusion dependent thalassemia patients and excluded β-thalassemia patients who were non transfusion dependent. However; the distinction between TDT and NTDT can be complex and may vary depending on factors such as the specific genetic mutations, geographic location, and individual patient factors. Some individuals with NTDT may eventually require transfusions as their condition progresses.

### Conclusion and recommendations

The present findings obviate the impact of met-haemoglobin on endothelial function in TDT patients so routine estimation of Met-Hb level should be done. As TDT patients are highly vulnerable to the effects of oxidative stress, serial calculation of EASIX is warranted being a simple biomarker of endothelial function. Antioxidant drugs should be supplemented to them.

## Data Availability

All data supporting the findings of this study are available within the paper and its Supplementary Information. All main data are available upon request. If someone wants to request data from this study you can contact the corresponding author: Maha Abubakr Feissal Rabie, Email: maha.feissal@pua.edu.eg, Mobile number: 0122230469, Fax number: 033877000. Address: Pharos University, Canal El Mahmoudia Street, beside Green Plaza Complex, Alexandria, Egypt.
